# Enhanced Transduction and Replication of RGD-Fiber Modified Adenovirus in Primary T Cells

**DOI:** 10.1371/journal.pone.0018091

**Published:** 2011-03-28

**Authors:** Sadhak Sengupta, Ilya V. Ulasov, Bart Thaci, Atique U. Ahmed, Maciej S. Lesniak

**Affiliations:** The Brain Tumor Center, The University of Chicago, Chicago, Illinois, United States of America; Cedars-Sinai Medical Center and University of California Los Angeles, United States of America

## Abstract

**Background:**

Adenoviruses are often used as vehicles to mediate gene delivery for therapeutic purposes, but their research scope in hematological cells remains limited due to a narrow choice of host cells that express the adenoviral receptor (CAR). T cells, which are attractive targets for gene therapy of numerous diseases, remain resistant to adenoviral infection because of the absence of CAR expression. Here, we demonstrate that this resistance can be overcome when murine or human T cells are transduced with an adenovirus incorporating the RGD-fiber modification (Ad-RGD).

**Methodology/Principal Finding:**

A luciferase-expressing replication-deficient Ad-RGD infected 3-fold higher number of activated primary T cells than an adenovirus lacking the RGD-fiber modification *in vitro*. Infection with replication-competent Ad-RGD virus also caused increased cell cycling, higher E1A copy number and enriched hexon antigen expression in both human and murine T cells. Transduction with oncolytic Ad-RGD also resulted in higher titers of progeny virus and enhanced the killing of T cells. *In vivo*, 35–45% of splenic T cells were transduced by Ad-RGD.

**Conclusions:**

Collectively, our results prove that a fiber modified Ad-RGD successfully transduces and replicates in primary T cells of both murine and human origin.

## Introduction

Adenoviruses attach to host cells via a cell surface receptor known as the coxackie and adenovirus receptors (CAR) [1], [2]. This attachment is augmented by binding with integrins on the host cell surface [3]. In 1995, Huang *et al.* first reported that activation of T cells increased the expression of α_v_β3 and α_v_β5 integrins on their cell surface and facilitated adenovirus mediated gene delivery [4]. Nevertheless, T cells are resistant to adenoviral infection because they lack the CAR needed for virus attachment [5], [6]. Several attempts have been made to transduce T cells with different recombinant adenoviruses, yet most of the existing literature is not clear about the method used by the virus to transduce in the absence of CAR [7]–[9]. It is known that RGD-fiber modified adenoviruses which have an RGD-motif inserted in the HI-loop of the fiber, use α_v_β3 and α_v_β5 integrins to attach to host cells [10], [11]. RGD-fiber modified viruses have been successfully used to transduce several hematological cells, especially dendritic cells [12]. Most of these studies were done with replication-deficient virus to deliver genes for immunomodulation. In this study, using a murine model, we show that RGD-fiber modified adenoviruses successfully transduce murine T cells and efficiently replicate in the transduced cells.

T cells can be potential targets for gene therapy for a variety of diseases including, but not limited to AIDS, hematologic malignancies, autoimmune disorders and transplantation rejection (reviewed by [13]–[15]). T cells have been targeted with retroviruses and lentiviruses for therapeutic gene delivery [16], [17]. These attempts were only partially successful because of low viral titers, their requirement of host cell-cycle progression to permit viral genome integration and most importantly their inefficiency in transducing T cells. On the other hand, adenoviral vectors are easy to produce in higher titers and contain a well characterized genome theoretically making them ideal for T cell transduction [18], [19]. Despite these advantages, studies that have investigated adenoviral transduction in T cells have been discouraging [8], [20]. These reports indicated that T cells required very high doses of infection and resulted in frustratingly low levels of efficiency. These obstacles have been attributed to the lack of CAR receptors on the surface of T cells [5], [6], [21]. To overcome CAR deficiency, several alterations have been attempted to improve adenoviral transduction of T cells. These include, modifying the fiber-shaft to bind to the CD3, replacing tail fibers on serotype 5 with that from 35 (Ad5F35) to target CD46, and using Lipofectamine during transduction [22]–[25]. All of these mechanisms have resulted in moderately successful increases in transduction efficiency. In addition, transgenic murine models have been developed to express hCAR receptors on T cells [21], [26]. However, physiologically these models are unrealistic because T cells do not express this receptor naturally. Hence, further research is warranted to identify an efficient method of adenoviral transduction of T cells.

The adenovirus wild-type fiber was modified with the introduction of an Arg-Gly-Asp (RGD)-containing peptide in the HI loop of the fiber knob domain resulting in the ability of the virus to utilize the α_v_β3 and α_v_β5 integrins during the cell entry process [10], [11]. This fiber modification has gained importance in adenovirus-mediated immune therapy because of the ubiquitous expression of α_v_β3 and α_v_β5 integrins on dendritic cells [27]. RGD-fiber modified adenoviruses (Ad-RGD) have been extensively used to transduce dendritic cells both of human hematopoietic origin and murine origin to express immunomodulatory factors for cancer immunotherapy [12], [27]–[29]. T cell activation induced expression of α_v_β3 and α_v_β5 integrins on cell surface has already been known for quite some time [4]. Although reports of successful RGD-fiber modified virus mediated gene delivery in T cells have been reported, knowledge about viral-replication in the transduced T cells has not yet been addressed [30].

Here, we investigated the efficiency of Ad-RGD virus to transduce T cells from mouse splenocytes and human PBMCs that were activated *in vitro*. We show that replication-deficient Ad-RGD viruses that express a luciferase gene transduce more efficiently than adenoviruses with wild-type fibers. In addition, oncolytic Ad-RGD viruses also replicate in the transduced T cells. This observation was supported by high E1A gene copy numbers, increased hexon protein expression, viral progeny titers and enhanced cytotoxicity of Ad-RGD transduced T cells. Treatment of experimental mice with Ad-RGD demonstrated higher infectivity of splenic T cells than virus with wild-type fibers. These data indicate that Ad-RGD adenoviruses transduce T cells and their replicative machinery is also efficiently utilized in the transduced cells.

## Materials and Methods

### Ethics Statement

Human tissue was collected in accordance with a protocol approved the Institutional Review Board (IRB) at the University of Chicago (#13779A). All participants signed a written consent. Animal experiments were performed according to a protocol (#71407) that was approved by the Institutional Committee on Animal Use at the University of Chicago.

### Animals

Six- to eight-week-old male C57BL/6 (B6) mice were purchased from The Harlan Sprague Dawley (Madison, WI). 2C TCR Tg Rag1^−/−^ transgenic mice were maintained as a heterozygous colony by crossing with C57BL/6 Rag1^−/−^ mice and screening for expression of 2C TCR on peripheral blood lymphocytes by flow cytometry with 1B2 clonotypic antibody. All mice were housed in a specific pathogen-free facility at the University of Chicago, and experiments were conducted according to federal and institutional guidelines and with the approval of the University of Chicago Institutional Animal Care and Use Committee.

### Reagents

Tissue culture reagents were obtained from Cellgro/Mediatech (Manassas, VA) and plastic ware from BD Biosciences (San Jose, CA). PCR and qPCR oligos were purchased from Integrated DNA Technologies (Coralville, IA). iScript cDNA synthesis kit was purchased from Bio-Rad Laboratories (Hercules, CA). SYBR-Greener RT-PCR mastermix was purchased from Invitrogen (Carlsbad, CA). Fluorescent-labeled antibodies for flow cytometry were purchased from eBioscience (San Diego, CA) or Millipore (Billerica, MA). All other reagents were purchased from Sigma-Aldrich (St. Louis, MO) and Thermo Fisher Scientific (Pittsburgh, PA).

### Recombinant adenoviruses and adenoviral constructs

The replication defective viruses AdWT-Luc, AdRGD-Luc, Ad5/3-Luc and AdPK7-Luc and the replication competent vectors AdWT and AdRGD viruses have been described previously [31]–[35]. The replication-deficient and competent (oncolytic) viruses were propagated in HEK293 (ATCC, Manassas, VA) and A549 cells (ATCC, Manassas, VA) respectively, purified by CsCl gradient and titrated in HEK293 cells [36].

### Activation of splenocytes and enrichment of CD8^+^ T cells

2C TCR Tg Rag 1^−/−^ mice were used as source of enriched CD8^+^ T cells. Splenocytes from 2C TCR Tg mice were activated with anti-CD3 monoclonal antibody (clone 145.2C11; 1 µg/ml) and cultured for 60 hrs in RPMI-1640 medium supplemented with 10% FBS, 50 µM β-mercaptoethanol, L-glutamine and antibiotics (R10F) and recombinant mouse IL-2 (rmIL2; 10 ng/ml; Peprotech, Rocky Hill, NJ) at 37°C in 5% CO_2_ atmosphere. Activated splenocytes were stained with CD8-PE and CD8^+^ T cells were enriched by FACS to 95–99% purity.

### Preparation of PBMC and enrichment of human CD8^+^ T cells

Five to ten milliliters of fresh blood was drawn from 3 human volunteers respectively according to University of Chicago Institutional review Board approved protocol. The drawn blood samples were diluted 1∶1 with PBS and layered over 3 ml of Lymphoprep™ (Axis-Shield, Norway) and buffy coat was separated according to manufacturer's protocol. The buffy coat was washed 3 times with PBS and resuspended in R10F supplemented with recombinant human IL-2 (rhIL2; 20 U/ml; Peprotech, Rocky Hill, NJ) @ 1×10^6^/ml. The cells were activated with phorbol myristate acetate (PMA; 50 ng/ml) and ionomycin (500 ng/ml) and CD8^+^ T cells were enriched from each sample by FACS.

### Infection of CD8^+^ T cells with adenoviruses

Fifty thousand enriched CD8^+^ T cells were plated in each well of a 96-well plate in R10F medium. The cells were adsorbed for 2 h by centrifuging at 1000 g with an MOI of 50 infectious units (iu) per cell of wild-type and recombinant adenoviruses expressing different fiber modifications [37]. The MOI was chosen from routine laboratory titrations. After adsorption, virus-containing medium was discarded and cells were washed twice with PBS and incubated with fresh R10F supplemented with IL2 for different time points at 37°C in 5% CO_2_ atmosphere.

### Infection of A549 human lung carcinoma cells with adenoviruses

A549 human lung carcinoma cells which are permissive for adenovirus transduction were tested in parallel to the murine T cell for transduction efficiency. In a typical experiment, 5×10^4^ A549 cells were plated in each well of a 24-well tissue culture plate in Dulbecco's Modified Eagles Medium supplemented with 10% FBS, L-glutamine and antibiotics (D10F). After overnight cultures, which allowed the cells to stabilize and adhere to the substratum, culture medium was replaced with D10F containing 50 MOI of respective replication-competent adenovirus. The cells were incubated with virus for 2 h at 37°C in 5% CO2 atmosphere for virus adsorption, following which the cells were washed twice with PBS and incubated for different time-points in D10F under normal tissue culture conditions.

### Transient transfection reporter assays

Activated murine T cells were transduced with a panel of replication-deficient recombinant adenoviruses with wild-type fiber or with different fiber modifications and expressing a luciferase gene. 50 MOI of each virus type was used for the study. Efficiency of adenovirus transduction was monitored by luciferase activity with the Luicferase Assay System (Promega, Madison, WI) in accordance with the manufacturer's protocol. Briefly, mock and virus transduced cells were harvested 48 h after adsorption and lysed in a lysis buffer. Lysed cells were added to the luciferase-substrate solution and luciferase activities were tested in a luminometer and are presented as RLU (relative light units) per mg of cellular protein. Each experiment was performed with at least six replicates.

### Quantitative Real-Time PCR

Activated murine or human T cells were treated with 50 MOI of oncolytic Ad-WT and Ad-RGD adenovirus as described above and incubated for different time-points up to 72 h. Virus replication in the transduced T cells was evaluated by studying relative changes of viral E1A expression after each time point. Total DNA was extracted from mock and virus-treated cells by DNeasy Blood and Tissue kit (Qiagen, Valencia, CA, USA) according to the manufacturer's instructions. Gene expression was quantified by quantitative PCR (qPCR) using SYBR GreenER PCR Master Mix (Invitrogen, Carlsbad, CA, USA) and primers recognizing E1A region as described previously [38]. The threshold cycle values for E1A were obtained from qPCR and converted to the gene copy number from the standard curves. DNA amplification was carried out using Opticon 2 system (Bio-Rad). All samples were performed in triplicate. The data are presented as E1A copy numbers per nanogram of total DNA of Ad-RGD to those of Ad-WT transduced T cells [36].

### Flow cytometry

Changes in expression of β3 and β5 integrins on primary murine T cells upon activation with anti-CD3 antibody were analyzed by flow cytometry. Briefly, naïve and anti-CD3 antibody treated splenocytes were stained for either CD4 or CD8 T cells with fluorescent dye-conjugated antibodies for respective markers and co-stained with anti-mouse CD61 (β3) and anti-human/mouse β5 antibodies conjugated with fluorescin-isothiocyanate (FITC). Anti-hamster IgG-FITC and anti-mouse IgG1κ-FITC were used as isotype controls for β3 and β5 respectively. The data were acquired by flow cytometry using BD FACSCalibur (BD Biosciences) and analyzed by FlowJo software (Version 8.8.6; Tree Star, Inc., Ashland, OR).

Mock, Ad-WT and Ad-RGD oncolytic virus transduced murine T cells and A549 human lung carcinoma cells were tested for hexon protein expression at 4, 16, 24, 48 and 72 h after treatment with virus. 50 MOI of each virus type was used for the study. Treated cells were fixed and permeabilized with ice-cold acetone-methanol for 15 min at 4°C and then incubated with anti-adenovirus-FITC antibody (Millipore, 1∶100) for 20 min at 4°C. Following incubation, the cells were washed twice with FACS buffer (PBS +1% BSA+0.02% NaN_3_) and expression of adenoviral hexon protein was analyzed by BD FACSCalibur. Data was represented as percentage of cells expressing hexon protein.

Viability of oncolytic virus transduced murine T cells was tested by annexin V/7-AAD exclusion method [39] using Annexin V apoptosis detection kit (BD Biosciences). Mock, Ad-WT and Ad-RGD oncolytic virus transduced murine T cells were harvested after 24, 48 and 72 h. Cells were resuspended in annexin-binding buffer and stained with annexin V-FITC for the detection of phosphatidylserine expression on the cell membrane of apoptotic cells, and counterstained with 7-amino actinomycin D (7-AAD) to test membrane integrity, for 15 min at room temperature. Reaction was stopped by diluting in 4x volumes of annexin-binding buffer and cell viability was analyzed by flow cytometry. Population of cells negative for both annexin V and 7-AAD was considered to be viable.

### Cell cycle assay

To demonstrate that adenovirus replicates in transduced T cells, we analyzed the cell cycle phases of the treated cells. T cells were treated with 50 MOI of oncolytic Ad-WT and Ad-RGD virus and harvested after 24, 48 and 72 h, respectively. Cells were washed twice with ice-cold PBS and fixed in ice-cold methanol. Fixed cells were rehydrated back with two washes of PBS and treated with RNase (100 µg/ml; Sigma) for 15 min at 37°C. Reaction was stopped by diluting in 3 volumes of PBS. Propidium iodide (50 µg/ml; eBioscience) was added to the RNase treated cells and PI dilution was analyzed immediately by flow cytometry.

### Virus progeny release

As a further evidence of virus replication in transduced T cells, viral progeny released from these cells was titrated. Culture supernatant and cell pellets were harvested from oncolytic Ad-WT and Ad-RGD transduced murine or human T cells at different time-points after treatment as described above. Cell pellets were lysed by subjecting them to repeated cycles of freeze-thaw. Viral titers were measured from cell-free (culture supernatant) and cell-associated (lysate) fractions of T cells on HEK293 cells using Adeno-X Rapid Titer Kit (Clontech, Mountain View, CA).

### Animal studies

To test whether adenovirus infects T cells *in vivo*, 2×10^7^ iu of Ad-WT or Ad-RGD virus was injected via tail vein in two separate groups of 6–8 weeks old C57/B6 mice [26]. A third group received only PBS and was designated as the Mock group. Three animals from each group were sacrificed at 0, 3, 6, 9, 16, 24, 72 and 96 h after injection and spleens were harvested. Single cell splenocyte suspensions were stained for CD4 and CD8 surface markers. The cells were then stained for hexon expression as described above. Populations of CD4^+^ and CD8^+^ T cells in the live gate that were expressing hexon antigens at each time point were analyzed by flow cytometry. The method for analyzing primary T cells in live gate has been described earlier [40].

### Statistical analysis

GraphPad Prism software package for Windows (version 4.0; GraphPad Software, La Jolla, CA) was used to run ANOVA, two-tailed unequal variance *t* test, Student's *t* test, standard deviation and standard error of measurement on the data presented. A value of *p*<0.05 was considered significant.

## Results

### β3 and β5 integrin expression on murine primary T cells

**Figure 1:**
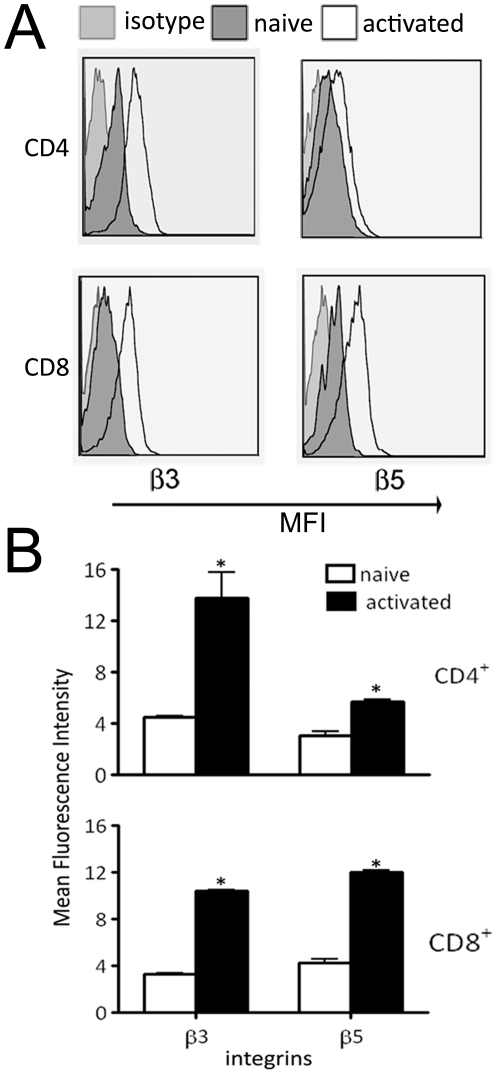
β3 and β5 integrin expression on primary murine T cells. (A) Flow cytometry profile of β3 and β5 integrin expression on naïve and activated primary murine T cells. *Left panel* shows histograms for β3 integrins on CD4^+^ *(top)* and CD8^+^ T cells *(bottom)*. *Right panel* shows β5 integrins on CD4^+^ and CD8^+^ T cells. (B) Bar diagram representation of β3 and β5 integrin expression on T cells. *Top panel* shows integrin expression on CD4^+^ T cells while *bottom panel* represents CD8^+^ T cells. *White bars* indicate naïve T cells and *black bars* are activated T cells. Error bars indicate mean ± SD. (*p<0.05)

Naïve and activated murine T cells were tested for the expression of β3 and β5 integrins on their surface. These integrins are components of cell-surface receptors for Ad-RGD viruses. Flow cytometric analysis showed 3.5-fold increase in mean fluorescence intensity of β3 and 1.5-fold increase in β5 expression on activated CD4^+^ T cells over naïve cells. In CD8^+^ T cells, 2.5-fold increase in both β3 and β5 expression was observed after activation with anti-CD3 antibody (Fig. 1).

### Mouse primary T cells can be transduced with RGD modified adenovirus

**Figure 2:**
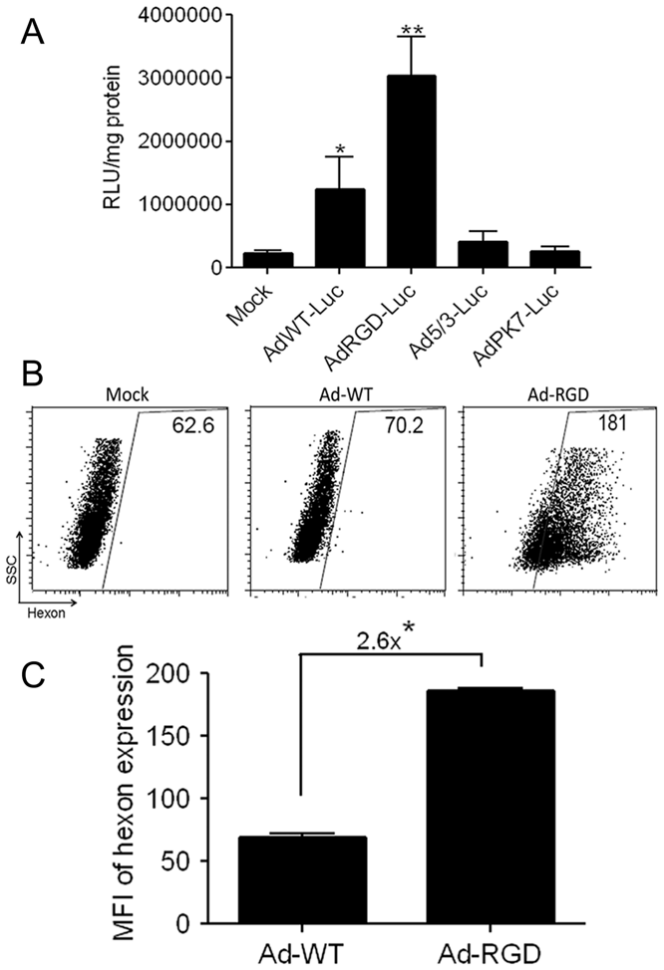
Primary murine CD8+ T cells can be transduced with RGD-modified adenovirus. (A) Primary mouse T cells were treated for 48 h with replication-deficient fiber-modified adenoviruses encoding luciferase gene. Luciferase activity was measured from lysates of treated cells and is represented as relative luciferase unit (RLU) per milligram of cell protein. Cells transduced with RGD-modified adenovirus (AdRGD-Luc) showed maximum luciferase activity. (*p<0.05) (**p<0.001). (B) Flow cytometric dot-plots showing mean fluorescence intensity of viral hexon proteins expressed by transduced T cells. Gates were drawn by excluding the mock-transduced cells (Mock) represented in the *left panel*. T cells that were transduced with oncolytic replication-competent adenovirus with wild type fibers (Ad-WT) are represented in the middle panel while cells transduced with RGD-fiber modified oncolytic adenovirus (Ad-RGD) are shown in *right panel*. (C) Bar diagrammatic representation of viral hexon MFI in adenovirus induced murine T cells. Ad-RGD transduced T cells expressed 2.6-fold more viral hexon proteins in comparison to Ad-WT transduced cells (*p<0.01). Error bars indicate mean ± SD.

To test the transduction efficiency of adenoviruses, activated CD8^+^ T cells were enriched from mouse splenocytes and were treated with a panel of replication-deficient recombinant adenoviruses with wild-type fiber or with fiber modifications, and expressing a luciferase gene. Forty-eight hours after virus treatment, the luciferase activity was measured from the treated cells upon incubation with a substrate as described in materials and methods. Maximum luciferase activity was observed in the cells that were transduced with RGD fiber modified adenovirus (AdRGD-Luc), which was appreciably higher than cells that were treated with adenovirus with wild-type fiber (AdWT-Luc). However, both of these treatments showed a statistically significantly higher luciferase activity than either mock or treatment with Ad5/3-Luc and AdPK7-Luc. This trend was observed in all the repetitions that were performed for this experiment (Fig. 2A).

We also tested transduction efficiency of replication-competent oncolytic adenoviruses with either wild-type fiber (Ad-WT) or RGD-modified fiber (Ad-RGD). Successful transduction of oncolytic Ad-RGD in T cells was established when adenoviral hexon proteins were expressed in cells at different-time points after virus adsorption. Flow cytometric analysis of viral hexon antigen expressed by the Ad-RGD treated T cells was 2.6-fold higher vs. Ad-WT treated cells (Fig. 2B, C).

### Ad-RGD replication in transduced T cells

**Figure 3:**
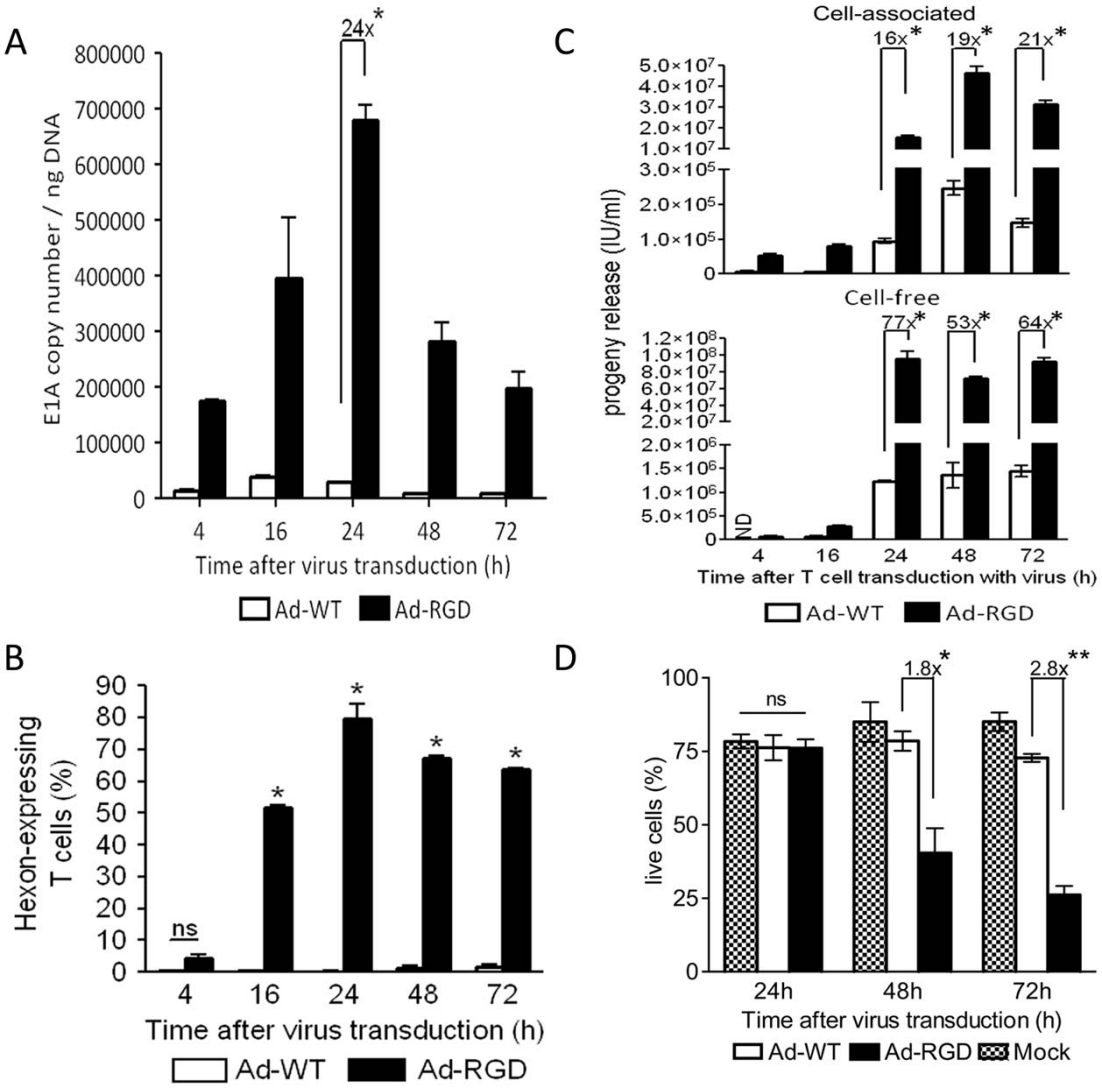
Evidence of adenovirus replication in Ad-RGD transduced T cells. (A) Increased adenoviral E1A gene copy number in Ad-RGD treated cells *(black bar)* in comparison to Ad-WT treated cells *(white bar)* at different time-points after virus transduction calculated by qPCR. 24-fold higher viral E1A gene copy number was observed in Ad-RGD treated cells versus Ad-WT transduced cells at 24 h time-point (*p<0.001). (B) Bar diagram of flow-cytometric analysis showing percentage of T cells expressing viral hexon antigen at different time-points after virus transduction. Ad-RGD transduced T cells are represented by *black bars* and Ad-WT treated cells by *white bars*. (*p<0.0001; *ns* not significant). (C) Viral progeny was measured from cell lysate *(cell-associated; upper panel)* and culture supernantant *(cell-free; lower panel)* of adenovirus infected T cells. 16- to 21-fold increase in viral progeny was measured in T cell lysates and 64- to 77-fold higher viral progeny was observed in cell-free fractions of Ad-RGD treated cells *(black bars)* when compared to cells treated with Ad-WT *(white bars*) at different time-points after virus transduction (p<0.001; *ND* not detected). (D) Viability of virus-transduced T cells was assessed by Annexin/7AAD exclusion method. Frequencies of Annexin/7AAD double negative cells observed in flow-cytometry were plotted in a bar diagram. 1.8-fold and 2.8-fold higher death of Ad-RGD transduced T cells *(black bars)* was observed at 48 and 72 h after virus transduction respectively, when compared to Ad-WT transduced T cells *(white bars)*. Mock *(patterned bars)* and Ad-WT showed similar viability through the entire time-course observation (*p<0.05; **p<0.001; *ns* not significant). Error bars represent mean ± SD.

To show that RGD-fiber modified adenoviruses not only transduce, but also replicate in mouse T cells, anti-CD3-activated T cells were treated with oncolytic Ad-RGD virus and tested for expression of viral components after different time-points. Oncolytic Ad-WT was used as control. Evidence of Ad-RGD viral replication efficiency was suggested by measuring viral E1A gene copy numbers by qPCR at 4, 16, 24, 48 and 72 h post-adsorption. We observed 1.7×10^5^ E1A copies/ng DNA in Ad-RGD treated cells at 4 h after virus treatment. The levels of E1A copy number in Ad-RGD transduced cells peaked at 24 h (6.8×10^5^ copies/ng DNA) which was 24-fold higher than observed in Ad-WT treated cells. In the following time-points studied, Ad-RGD E1A copy numbers were reduced to 2.5×10^5^ and 1.8×10^5^ copies/ng DNA at 48 and 72 h, respectively. In comparison, these numbers were still significantly higher than those of Ad-WT virus, which failed to replicate efficiently in murine T cells (Fig. 3A). This further asserted the non-permissiveness of these cells to adenoviruses with wild-type fibers.

**Figure 4:**
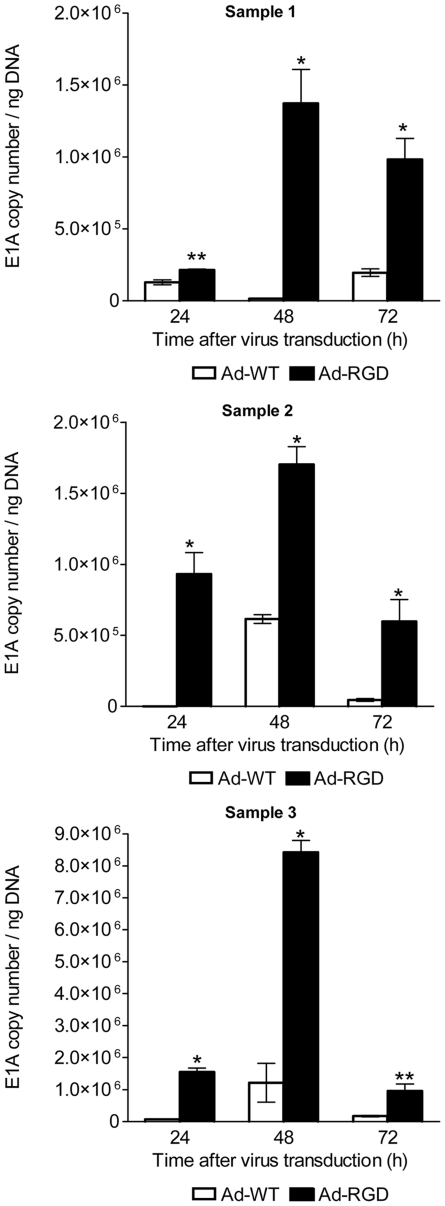
Adenoviral E1A copy numbers in transduced human CD8^+^ T cells. Increased adenoviral E1A gene copy number in Ad-RGD treated cells *(black bar)* in comparison to Ad-WT treated cells *(white bar)* at different time-points after virus transduction calculated by qPCR. Samples 1–3 are three independent experiments. (*p<0.001; ** p<0.05). Error bars represent mean ± SD.

Similar results were observed when primary human CD8^+^ T cells were treated with Ad-WT and Ad-RGD. E1A copy numbers of Ad-RGD virus were significantly higher than Ad-WT as early as 24 h post-transduction and increased even more by 48 h (Fig. 4).

However, the ability of both Ad-WT and Ad-RGD viruses to replicate in A549 cells was more efficient than murine T cells. E1A copy numbers of both viruses obtained from A549 cells were several magnitudes higher than those from murine T cells (Supplementary Figure S1A).

Kinetics of Ad-RGD replication was further analyzed by measuring viral hexon protein in transduced T cells. Although hexon antigen was observed as early as 4 h after virus treatment, there was no difference in the percentage of T cells that were transduced with either Ad-WT or Ad-RGD treated cells. However, 16 h after transduction, 50% and by 24 h almost 80% of the Ad-RGD transduced T cells were hexon-positive and this trend was continued at the later time-points studied. In comparison, less than 1% of the Ad-WT transduced T cells were hexon-positive for all of the time-points studied (Fig. 3A, B). The levels of hexon expression by both Ad-WT and Ad-RGD viruses were very similar in A549 cells and by 24 h post-transduction, more than 80% of both the virus-treated groups were hexon-positive (Supplementary Figure S1B).

### Higher viral progeny titer from Ad-RGD transduced T cells

The efficiency of Ad-RGD viral replication was confirmed by titration of the viral progeny released from the transduced T cells. This method has been often used as a measure of viral replication [41], [42]. Viral progenies were measured in the cell-free and cell-associated fractions of Ad-WT and Ad-RGD transduced mouse T cells that were harvested after 4, 16, 24, 48 and 72 h of treatment. Titration of viral progeny in respective fractions from each time-point was performed on HEK293 cells. Results indicated a 16-fold higher viral progeny in cell-associated fractions of Ad-RGD treated cells compared to Ad-WT treated cells that were harvested as early as 24 h after T cell incubation. The viral progeny titers were further increased by 19- and 21-fold over Ad-WT progenies at 48 and 72 h, respectively. In cell-free fractions (T cell culture supernatants), however, a 77-fold higher viral titer was obtained from Ad-RGD treated cells when compared to that of Ad-WT treated cells at the 24 h time-point. This trend was maintained in the cell-free fractions from two time-points at 48 and 72 h (Fig. 3C). At earlier time-points, no significant difference in viral progenies of both Ad-WT and Ad-RGD was observed.

**Figure 5:**
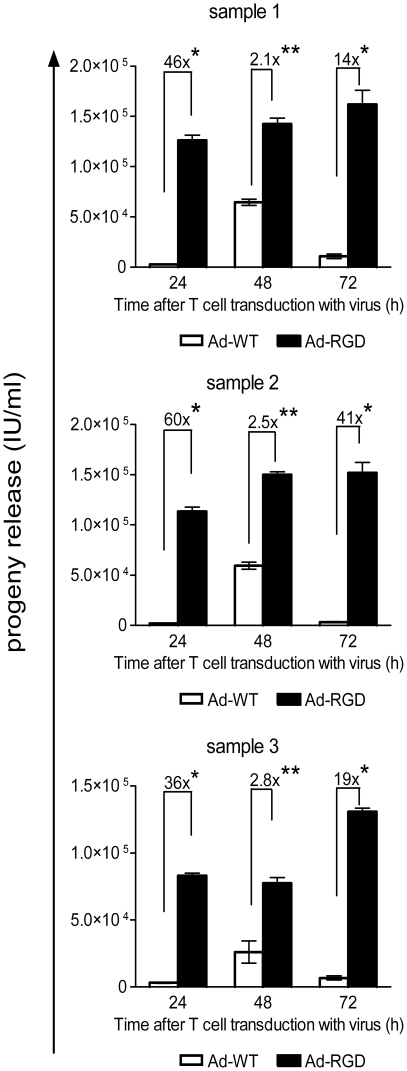
Viral progeny release from transduced human CD8^+^ T cells. Viral progeny was measured from culture supernantant of adenovirus infected human T cells. 36- to 60-fold increase in viral progeny was measured in supernatants of T cell lysates treated with Ad-RGD *(black bars)* when compared to cells treated with Ad-WT *(white bars*) at 24 h after virus transduction. Samples 1–3 are three independent experiments (*p<0.001; ** p<0.01). Error bars represent mean ± SD.

Human primary T cells yielded a 36 to 60-fold higher viral progeny when transduced by Ad-RGD in comparison to Ad-WT as early as 24 h post-transduction. The difference was reduced at 48 h but increased viral progeny from Ad-RGD transduced cells was observed again after 72 h after transduction (Fig. 5).

### Viability of Ad-RGD transduced T cells

Increasing cytotoxicity of Ad-RGD treated T cells was also a proof of viral replication in activated murine T cells. Viability of virus and mock-treated T cells was analyzed by annexin V/7-AAD exclusion method and by flow cytometry. At 24 h after infection, although there was no difference in viability of cells between the two-treatment groups, 1.8-fold more Ad-RGD treated T cells were dead. By 72 h less than 25% of the Ad-RGD treated cells were alive, which was 2.8-fold less than the Ad-WT treated cells. However, more than 75% of the mock treated cells were alive at all the time-points studied. (Fig. 3D)

### T cell transduced with oncolytic Ad-RGD undergo rapid cell cycle progression

**Figure 6:**
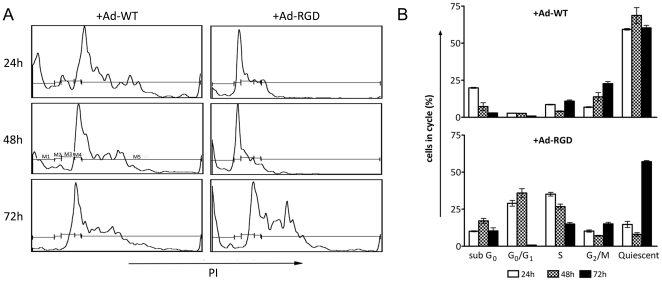
T cells transduced with oncolytic Ad-RGD undergo rapid cell cycle. (A) Flow cytometric profile of propidium iodide (PI) dilution in virus-transduced T cells at different time-points after treatment. Gates drawn on different cell-cycle phases are *M1* sub-G0, *M2* G0/G1, *M3* S, *M4* G2/M and *M5* quiescent. *Left panel* shows cells treated with adenovirus with wild-type fiber (Ad-WT). *Right panel* shows cells with RGD-modified oncolytic virus (Ad-RGD). (B). Bar diagram representation of the cell-cycle. *Top panel* represents cells treated with Ad-WT. *Bottom panel* represents cells treated with Ad-RGD. *White bars* are cells after 24 h, while *patterned* and *black* bars are cells after 48 and 72 h respectively after virus transduction. Error bars represent mean ± SD.

We performed a cell cycle analysis of T cells that were treated with oncolytic Ad-WT, Ad-RGD viruses to test the replication efficiency in the transduced cells. Virus treated cells were stained with propidium iodide to evaluate their cell cycle phases by flow cytometry. Propidium iodide binds to the cellular DNA and the intensity of propidium iodide indicates the levels of DNA in a particular phase of the cell cycle. The results showed that 25–30% of the Ad-RGD treated cells were in G_0_/G_1_ and S-phase respectively at 24 h and 48 h after virus treatment while 60–70% of the Ad-WT treated T cells were quiescent during the entire study period. At 72 h, most of the Ad-RGD treated cells were out of G_0_/G_1_ and S phase and accumulated in the quiescent phase, which corroborates with viability results where more than 67% of these cells were dead or dying (Fig. 6). Since virus utilizes the cell's cycling machinery to replicate, this rapid cycling of Ad-RGD transduced T cells supports the notion that the virus replicates in the cells.

### Efficient transduction of mouse T cell by Ad-RGD virus *in vivo*

**Figure 7:**
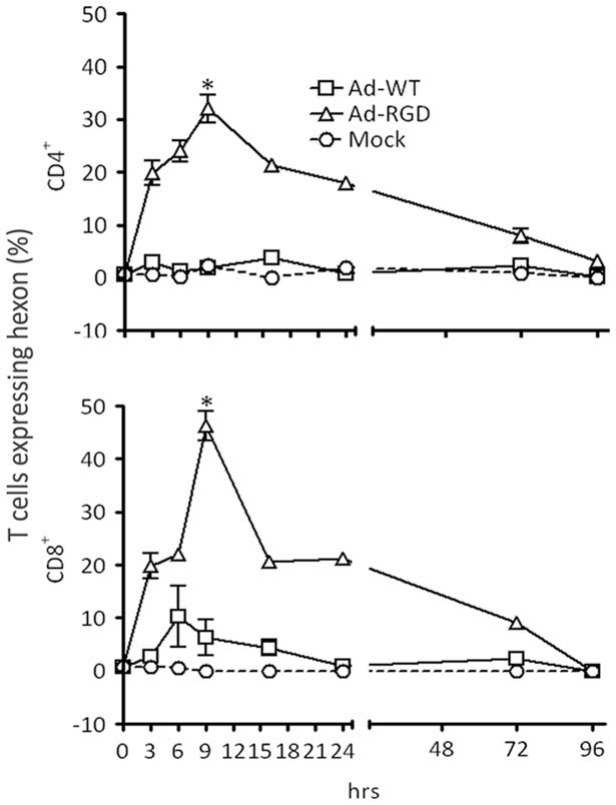
Efficient *in vivo* transduction of mouse T cells by Ad-RGD. Three groups of B6 mice were injected via tail-vein with Ad-WT *(open squares)*, Ad-RGD *(open triangles)* and PBS *(open circles)* respectively. Animals were sacrificed at 0, 3, 6, 9, 16, 24, 72 and 96 h after virus injection and spleens were harvested. Splenocytes were surface stained for CD4 and CD8 and then fixed and permeablized and stained with anti-hexon antibody for flow-cytometric analysis of adenovirus infected splenic T cells. Percentage of CD4^+^ T cells expressing adenoviral hexon antigen is represented in *upper panel* and CD8^+^ T cells are in *lower panel*. Error bars indicate standard deviation from three animals per group of study. (*p<0.01;**p>0.05; N = 3)

To test whether transduction of T cells by Ad-RGD could be replicated *in vivo*, B6 mice were injected with either PBS (Mock) or Ad-WT or Ad-RGD virus. Animals were sacrificed at different time-points after treatment. Splenocytes were stained for CD4 and CD8 and both the populations were counterstained for intracellular expression of viral hexon proteins. CD4^+^ and CD8^+^ T cells that were present in the live gate of forward-scatter/side-scatter flow cytometric dot plot were analyzed for hexon expression. Significantly higher hexon staining was observed in mice from Ad-RGD treated group, as early as 3 h after injection, when compared to Ad-WT treated mice. Nearly 35% of splenic CD4^+^ and 45% of CD8^+^ T cells from Ad-RGD treated mice were hexon-positive after 9 h of virus injection. However, these high levels were transient, and by 16 h the numbers came down to 20% in both CD4 and CD8 populations, but higher hexon expression was observed in T cells of Ad-RGD treated mice after 72 h of virus injection. The T cells from Ad-WT treated group showed minimal hexon expression and followed the same pattern as T cells from mock-treatment animal group (Fig. 7).

## Discussion

The observations in this study are remarkable because they show (1) that murine T cells can be transduced with human Ad-RGD virus and (2) that transduced virus can efficiently replicate in the T cells.

We chose to use the murine system primarily because of the availability of a T cell repertoire that can be easily manipulated both *in vivo* and *ex vivo*. Additionally, the investigation of adenovirus replication in a murine system has yet to be studied. CD8^+^ T cells were chosen for most of our experiments, based on observation of equally high increase in expression of both β3 and β5 integrins upon activation with anti-CD3 antibody (Fig. 1).

RGD-fiber modified (Ad-RGD) virus showed maximum transduction efficiency when activated T cells were incubated *ex vivo* with a panel of replication-deficient adenoviruses with different types of fiber modifications to deliver a luciferase gene (Fig. 2A). These results support the recent report by Ye *et al*, [30] who transduced CTLs with replication-deficient Ad-RGD to deliver TNF-α gene for immunotherapy of lung tumors. However, in another experiment, where murine T cells were exposed to oncolytic replication-competent Ad-RGD virus, higher levels of viral hexon proteins were expressed when compared to those of Ad-WT virus (Fig. 2B, C). This further confirmed successful transduction efficiency of Ad-RGD virus in murine T cells.

We aimed our study to examine the replication of the virus in the transduced T cells. We chose onolytic Ad-WT, with wild-type fiber and Ad-RGD. Our laboratory and others have regularly used the method of measuring viral E1A copy number as a marker for viral replication [36], [38]. Not only did Ad-RGD transduce better in murine T cells, kinetics of viral E1A gene copy number showed a 24-fold increase over Ad-WT in the transduced cells (Fig. 3A). Hexon protein expression in transduced cells is also used to measure viral replication [43]. Therefore, we further tested the viral replication efficiency in murine T cells by measuring the levels of adenoviral hexon expression. Higher percentages of Ad-RGD treated cells expressed the viral hexon protein than Ad-WT treated cells, confirming the replicative property of Ad-RGD in murine T cells (Fig. 3B).

The most compelling evidence of viral replication in the transduced T cells was demonstrated in the viral progeny studies (Fig. 3C). Cell-associated fraction, i.e., cell lysates of Ad-RGD transduced T cells isolated between 24 and 72 h of transduction produced 16- to 21- fold higher viral progeny vs. Ad-WT treated cells. The Ad-RGD viral progeny titers were further enhanced to 77-fold over Ad-WT when cell-free fractions from 24 h time-point were measured. This proves that viral replication in transduced cells depends upon the efficiency of the virus to enter the host cells, as AD-WT virus failed to replicate because of poor transduction efficiency.

We observed similar transduction effect of Ad-RGD virus in human T cells isolated from PBMCs of healthy donors. Expression of high viral E1A copy numbers and 36 to 60-fold increased viral progeny from Ad-RGD transduced T cells over those treated with AD-WT confirmed that this transduction efficiency effect was not host-species specific (Fig. 4 and 5). However, the total progeny yield from Ad-RGD transduced human T cells was very low. This observation could be explained by previous reports from Gooding's group, where the authors have described a post-internalization latency in viral reproduction in T cells isolated from tonsils of infected patients as well as human T cell lines [44]–[46].

Ad-RGD transduced T cells also cycled faster than the Ad-WT transduced cells as evident from the cell cycle assay. Because replicating virus utilizes host cell's replication machinery to propagate [18], we hypothesized that Ad-RGD stimulated the host cells to enter S phase of the cell cycle to provide an optimal intracellular environment for viral reproduction. This hypothesis was also partly based on our earlier observations of higher E1A copy numbers and hexon protein expression in these cells. Cell cycle analysis confirmed our hypothesis. Ad-RGD transduced T cells were found mostly in the S- and G_0_/G_1_ phase after 24 h of treatment, while the Ad-WT cells were mostly quiescent (Fig. 6). Seventy-two hours after treatment with Ad-RGD, the cells were released from the S phase and there was an increase in quiescent phase which suggested that most of the transduced cells were dead by this time.

To test virus-induced cell death, we tested the viability of virus-transduced T cells. Ad-RGD oncolytic virus enhanced the death of T cells upon successful transduction. At 72 h post-treatment, more than 75% of the Ad-RGD transduced cells were dead while the >75% of Ad-WT treated cells were still viable (Fig. 3D). This observation also confirmed that oncolytic Ad-RGD virus were successful in replicating and propagating inside transduced murine T cells.

Our *in vivo* observations also corroborated with our *ex vivo* results. We treated a cohort of C57/B6 mice with either Ad-WT or Ad-RGD virus following a regimen of *in vivo* virus injection reported previously [26]. PBS was injected in the mock-treatment group. Starting 3 h after injection, a higher percentage of both CD4^+^ and CD8^+^ splenic T cells that were injected with Ad-RGD virus started expressing viral hexon proteins. Maximum transduction efficiency was observed after 9 h of injection following which there was rapid loss of hexon-epressing T cells, which was probably due to rapid viral clearance. Ad-WT injected mice, however, failed to show any hexon protein in the splenic T cells which was similar to the PBS-treated animals (Fig. 7). This observation confirms that RGD-fiber modified recombinant adenoviruses are capable of infecting murine T cells *in vivo*.

Collectively, this study shows for the first time that human adenoviruses can not only successfully transduce T cells, they can also efficiently replicate in the transduced cells. However, the transduction efficiency is strictly limited to the recombinant adenoviruses that have been modified to express the RGD-motif on the viral fiber. This RGD-fiber modification utilizes increased β3 and β5 integrin expression in activated T cells to attach and enter the host cells. This study along with our recent report of cotton rats [47], [48] as semi-permissive hosts of human adenoviruses concludes that if adenoviruses can gain access they can propagate in any host species or cell type. It is significant because it indicates a possible use of adenovirus-mediated gene delivery for hematological malignancy research. Also, in light of the speed and efficiency of adenovirus transduction, T cells can be used as cell carriers to deliver adenovirus for gene therapy.

## Supporting Information

Figure S1 **Replication efficiency of Ad-WT and Ad-RGD in permissive human lung carcinoma A549 cells.** (A) Adenoviral E1A gene copy number in Ad-RGD treated cells *(black bar)* in comparison to Ad-WT treated cells *(white bar)* at different time-points after virus transduction calculated by qPCR. (B) Bar diagram of flow-cytometric analysis showing percentage of A549 cells expressing viral hexon antigen at different time-points after virus transduction. Ad-RGD transduced A549 cells are represented by *black bars* and Ad-WT treated cells by *white bars*. Error bars represent mean + SD.
(TIF)
