# Supplementary figures and images for: Enhanced Transduction and Replication of RGD-Fiber Modified Adenovirus in Primary T Cells
Source: PLoS One. 2011 Mar 28;6(3):e18091. doi: 10.1371/journal.pone.0018091 (PMC3065494; doi:10.1371/journal.pone.0018091)

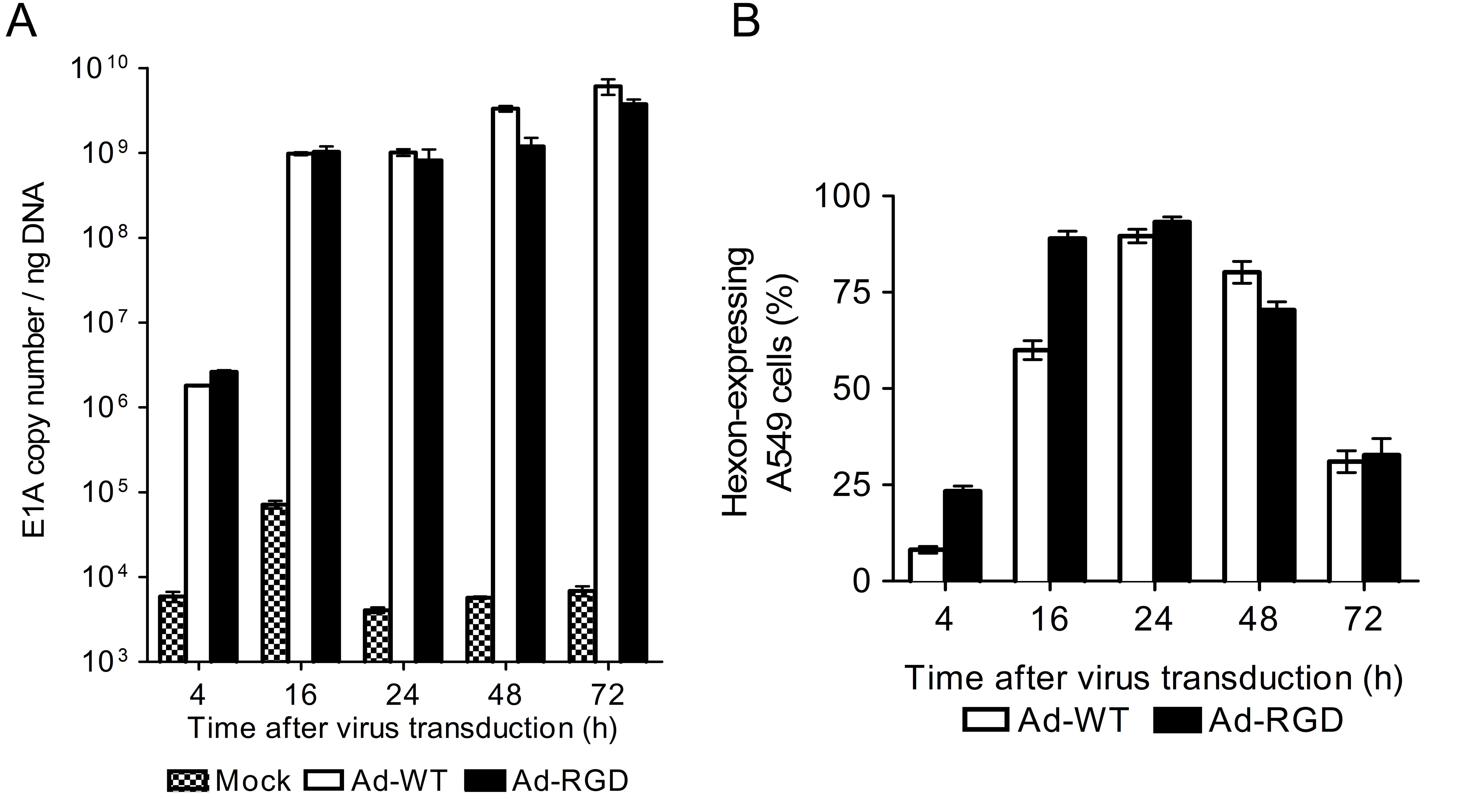

Supplement: Figure S1 — Replication efficiency of Ad-WT and Ad-RGD in permissive human lung carcinoma A549 cells. (A) Adenoviral E1A gene copy number in Ad-RGD treated cells (black bar) in comparison to Ad-WT treated cells (white bar) at different time-points after virus transduction calculated by qPCR. (B) Bar diagram of flow-cytometric analysis showing percentage of A549 cells expressing viral hexon antigen at different time-points after virus transduction. Ad-RGD transduced A549 cells are represented by black bars and Ad-WT treated cells by white bars. Error bars represent mean + SD. (TIF) [file pone.0018091.s001.tif]
